# Language Preference and its Moderating Role in Coping with Stress: The Hispanic Community Health Study/Study of Latinos

**DOI:** 10.18103/mra.v11i10.4625

**Published:** 2023-10-26

**Authors:** Morgan Gianola, Maria M. Llabre, Linda C. Gallo, Martha L. Daviglus, Daniela Sotres-Alvarez, Neil Schneiderman

**Affiliations:** 1 University of Miami, Department of Psychology; 2 San Diego State University, Department of Psychology; 3 University of Illinois, College of Medicine; 4 University of North Carolina, Department of Biostatistics

## Abstract

Stress and stressful events are widely accepted risk factors for cardiometabolic diseases, including coronary heart disease and diabetes. As language plays a seminal role in development and regulation of emotions and appraisals of stressful situations, it may contribute to documented differences in the stress-cardiometabolic disease association across ethnic groups. We investigated associations between language preferences (Spanish vs English) and downstream health consequences of stress. Using data from the Sociocultural Ancillary Study of the Hispanic Community Health Study/Study of Latinos, we assessed the relationship between reported stress and risk factors (alcohol use, smoking, body mass index, depressive symptoms) and prevalence of self-reported (coronary heart disease, stroke, chronic obstructive pulmonary disease [COPD]) and clinically assessed chronic conditions (diabetes, hypertension) among 5154 Hispanic/Latino adults living in the US. Factor analysis was used to calculate a composite stress variable from participants’ self-reported chronic stress, perceived stress, and adverse childhood experiences. Sampling weights and survey methodology were integrated in all analyses to account for this study’s complex survey design. After controlling for sociodemographic factors (Hispanic/Latino background, study site, years in the US, social acculturation, education, income, age, sex), higher composite stress scores were associated with elevated risk factors and greater prevalence of coronary heart disease, diabetes, and COPD. Furthermore, the relationship between stress and COPD was significantly stronger among Hispanic/Latino adults who preferred to be interviewed in Spanish (compared to English). Stronger connections between stress and likelihood of drinking alcohol among English-preferring persons also emerged. These results are interpreted in light of the Hispanic health paradox and the role of cultural processes in the development of health risk factors and chronic conditions. Our findings can be integrated into relevant approaches to address health disparities within and across Hispanic/Latino populations in the US.

## Introduction:

Effective strategies to address health disparities across racial and ethnic groups should reflect variation in the complex relationships between stress, disease, and wellness within and across communities from diverse cultural backgrounds. This study addresses whether the strength of the stress-health relationship for several chronic conditions and risk factors differs according to language preferences (Spanish vs English) in a diverse Hispanic/Latino population in the US. We first consider established associations between several domains of stress and health indicators as they relate to the complex health profile of this population. We then reflect on language as a highly salient cultural feature which may influence stress reactivity and downstream health consequences. Using data from the Sociocultural Ancillary Study of the Hispanic Community Health Study/Study of Latinos (HCHS/SOL), this investigation draws attention to the role of language in previously described stress-cardiometabolic disease associations^1^ (see for a review^2^). Understanding how language preference may influence the range of health consequences associated with stress exposure within the diverse Hispanic/Latino population is vital to remedying well-documented health and treatment disparities in the US (for example^3^).

A wide variety of stress indicators have been linked to health within the US population and among Hispanic/Latino adults specifically. For example, chronic stress associated with low socioeconomic status has long been tied to prevalence of coronary heart disease (CHD), total cardiovascular diseases (CVD), and stroke^4,5^. Similarly, chronic and perceived stress indicators relate to hypertension incidence^6^, angina and worse general health after acute myocardial infarction^7^, risk of stroke^8^, and secondary ischemic events after minor stroke^9^; impaired stress recovery is further associated with adverse general cardiovascular outcomes^10^. Adverse childhood experiences (ACE) likewise predict risk factors such as body mass index (BMI) and smoking^11^ and relate to population levels of physical health problems including asthma, obesity, and inflammation^12^. Specifically in the HCHS/SOL, Gallo, Roesch, et al.^1^ reported positive associations between chronic stress and prevalence of CHD, diabetes, and hypertension, whereas measures of traumatic and perceived stress were linked to higher likelihood of smoking. Relatively high ACE prevalence among Hispanic/Latino adults in the US was also associated with BMI, depressive symptoms, smoking, chronic obstructive pulmonary disease (COPD), and CHD^13^. Importantly, distinct stress indicators (e.g., chronic stress, ACE) are often highly correlated or predictive of each other^11,14^, suggesting that these indicators could be integrated into an underlying latent “general stress” variable to better capture the broad connections between stress and health. This approach was taken in the present investigation in order to extend prior findings related to the stress-disease relationship.

Stress may interact with several cultural processes to contribute to the “Hispanic Paradox”, findings of lower overall prevalence and mortality rates of multiple cardiovascular diseases among Hispanic/Latino adults in the US despite worse risk factor profiles than non-Hispanic Whites^15-17^. However, recent work calls into question the veracity of this paradox^18^. Several features of Hispanic/Latino culture may influence appraisal of and reactivity to the host of acute and chronic stressors associated with immigration and acculturation (see for a review^19^). Strong adherence to traditional Hispanic/Latino culture is hypothesized to be protective against negative health risks^20^, with sympathy, familism, and harmonic relationships noted as favorable cultural characteristics linked to better physical and emotional well-being^17^. For example, higher levels of psychosocial stress counterintuitively predict lower 10-year CVD risk, which the authors hypothesize relates to greater willingness to turn to social support systems among those reporting higher stress, leading to mitigation of negative health effects of stress^21^. Other researchers further propose that sociodemographic factors (e.g., generational and socioeconomic status, bilingual competence) can meaningfully influence social factors such as household size, family structure, and availability of emotional support, in turn altering susceptibility to the negative health consequences of stress^16,22,23^. Ruiz et al.^24^ posit a culturally tailored stress model in which downstream psychophysiological effects of high stress exposure are buffered by sociocultural factors moderating Hispanic/Latino stress appraisals. This framework suggests that the relationship between stress and health risk factors and outcomes may vary based on relative preservation of protective features of Hispanic/Latino culture.

Language is a central cultural feature previously tied to health risk factors and outcomes, as individuals and families maintain the Spanish language or adopt English as their preferred language. To illustrate, Guerra et al. report that greater Spanish proficiency is linked to lower odds of insufficient physical activity, which they contrast against higher odds of obesity for Hispanics/Latinos who express greater US-American cultural identity^25^. Lower smoking rates among study participants with higher Spanish compared to English usage^26^ have similarly been interpreted as acculturation effects and subsequently linked to studies tying measures of US-American acculturation to several CVD risk factors^27^. While such results tie language-based acculturation to several health relevant outcomes, limitations and inconsistent interpretation of language proficiency and acculturation metrics across public health studies have been noted^28^. Thus, alternative approaches which avoid treating language usage solely as a proxy for acculturation may improve understanding of factors predicting health behaviors and wellness.

Beyond its connection to broader acculturative processes, language itself has been shown to significantly influence stress and emotion processing. While expressive language plays a central role in development of emotion regulation^29^, the Spanish language presents a wider range of emotional and positive words than English (and several other languages)^30^, indicating that features of Spanish could enhance emotional expression and influence stress appraisal in a health protective manner^31^. Such effects are exhibited within bilingual individuals across languages. For example, emotional and self-bias word processing effects emerge more strongly in Spanish compared to English among bilingual native Spanish speakers^32,33^. Responses to experimentally induced pain have also been shown to vary across English and Spanish speaking contexts, according to bilingual participants’ preference for Hispanic/Latino versus US-American culture^34^. These patterns of emotion processing may impact stress reactivity specifically, as English-preferring Hispanic/Latino individuals experience more physical stress from racial microaggressions, associated with poorer self-rated health, than their Spanish-preferring counterparts^35^. Furthermore, Jimenez et al.^36^ found that Spanish preferring older Hispanic/Latino adults, despite showing higher prevalence and intensity of pain than English preferring Hispanic/Latino and non-Hispanic White adults, reported significantly lower functional limitation from pain, underscoring the Spanish language’s potential to buffer against health consequences of certain stressors. Taken together, these findings highlight the Spanish language’s potential to influence stress appraisal and downstream health behaviors and outcomes, while highlighting language preference as a generally stable indicator linked to stress responses, risk behaviors^25,26,37-39^, and health outcomes within the Hispanic/Latino population. To disentangle language effects on stress processing from general acculturation, this investigation thus incorporates separate indicators of language preference (dichotomized as English or Spanish) and a continuous indicator of social acculturation.

**Figure 1:**
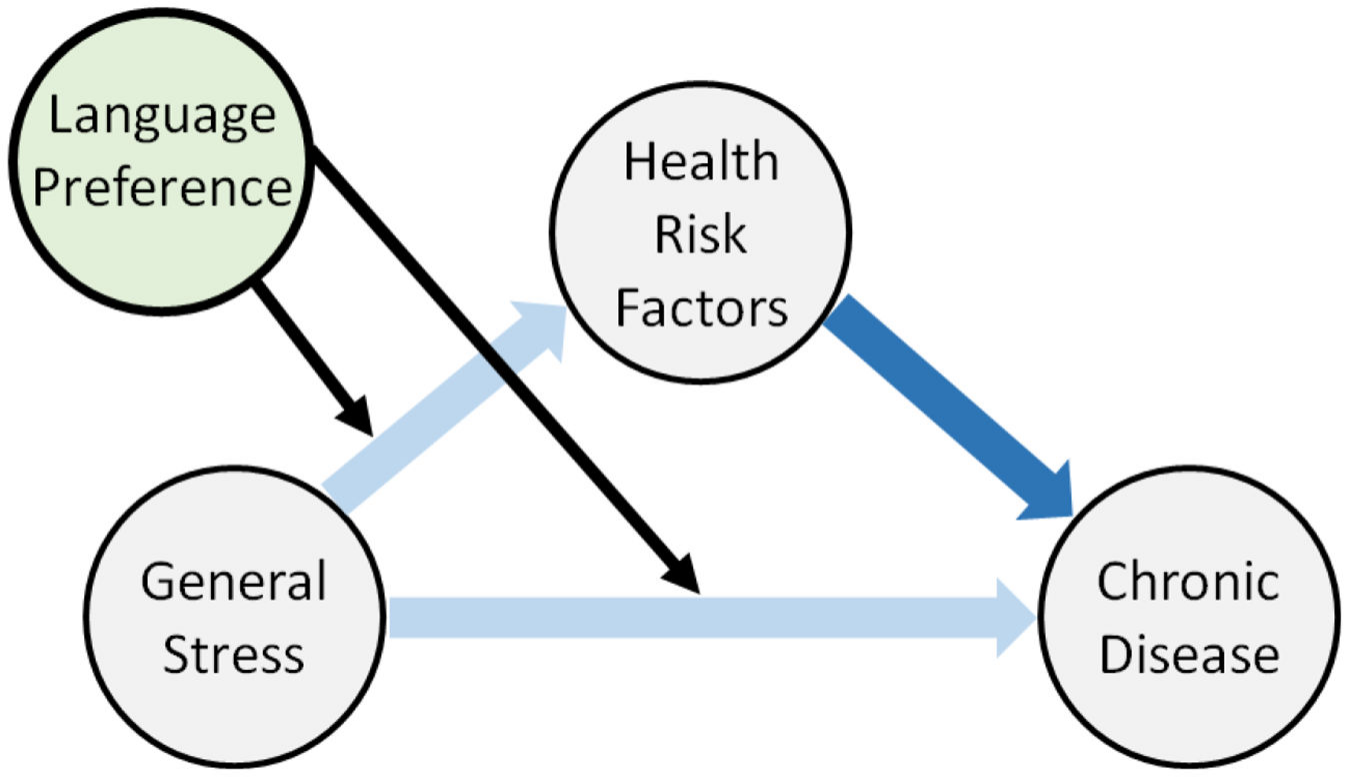
Conceptual model diagram. We hypothesize stress will predict chronic disease states and relevant health risk factors (light blue arrows) to different degrees across individuals who prefer English vs Spanish (black arrows). Chronic disease models control for behavioral risk factors (dark blue arrow) to assess whether language preference directly impacts the stress to chronic disease relationship.

The present analyses build from previous work demonstrating connections between distinct measures of stress and various health outcomes in the HCHS/SOL population^1,13,40,41^, by assessing the variability of this relationship across persons with distinct language preferences. Specifically, we compare participants who elected to respond in Spanish versus English on sociodemographic, risk factor, and stress variables (chronic stress, perceived stress, ACE) recorded at Visit 1. We calculate a composite “general stress” variable to assess whether broadly defined stress effects on risk factors and/or chronic disease prevalence are moderated by language preference (Figure 1). Controlling for sociodemographic variables, we test for language preference and stress effects on BMI, smoking, alcohol use, and depressive symptoms in the “risk factor models”. The “chronic disease models” then control for sociodemographic and risk factor variables to test for language preference and stress effects on self-reported (CHD, stroke, COPD) and clinically assessed chronic diseases (diabetes and hypertension) recorded approximately six years later at Visit 2. We test for effects of both self-reported and clinically verified diseases, as differences in reporting styles could confound observed relationships with self-reported health outcomes^42^, though some work suggests self-report sensitivity may not be as low for prevalence as compared to incidence estimates^43,44^. This investigation extends prior research showing fewer cardiometabolic risk factors and lower prevalence of CHD and stroke among Spanish responding participants in the full HCHS/SOL sample at Visit 1^45^. We attempt to isolate language preference from other acculturative processes by controlling for an indicator of social acculturation as well as years in the US in all models. Deeper understanding of the role of language in stress reactivity and health outcomes could contribute to culturally sensitive approaches to study and redress health disparities across Hispanic/Latino communities.

## Methods:

### DATA COLLECTION AND PARTICIPANTS

The sample selection and data collection procedures of the Hispanic Community Health Study/Study of Latinos (HCHS/SOL) have been described previously^46,47^. Briefly, HCHS/SOL is a multicenter prospective cohort study seeking to assess prevalence, incidence, and risk and protective factors for various chronic conditions among Hispanic/Latino adults living in the US. Probability sampling was conducted in three stages in four field centers: Bronx, NY; Chicago, IL; Miami, FL; and San Diego, CA. Census blocks were first randomly sampled then households were randomly selected within census blocks. Older participants (45-74 years old), Hispanic/Latino neighborhood concentration, and proportion of high socioeconomic status were over sampled. At Visit 1 (2008 to 2011), 16415 participants between 18 to 74 years old were enrolled. The Visit 1 examination consisted of an interview for sociodemographic information and behavioral measures and a clinical examination including anthropometric assessment, electrocardiogram, scan of prescribed medications, and other procedures. Visit 2 (approximately 6 years later; 2014 to 2017) included a clinical examination and interview. The present analyses incorporated data from Visits 1 and 2 from participants that also took part in the Sociocultural Ancillary Study (2010 to 2011), comprising 5313 adults, age 18-74, of self-identified Hispanic/Latino descent from a diversity of background groups: Mexican, Puerto-Rican, Cuban, Central American, Dominican, South American, and other/mixed backgrounds. All procedures were approved by institutional review boards from all participating institutions of the HCHS/SOL and Sociocultural Ancillary Study, and written informed consent was obtained from all participants.

### MEASURES

Data from Visit 1 were used for each participant’s demographic and risk factor variables. All demographic characteristics were self-reported during the interview; questionnaires were staff-administered by bilingual staff who were centrally trained. Demographic control variables included age, sex, Hispanic/Latino background, level of education, household income, health insurance status, and years living in the US. Language preference (Spanish or English) was defined based on the language the participant selected for their HCHS/SOL interviews. Risk factors included body mass index (BMI), alcohol use, smoking behavior, and depressive symptoms. BMI was calculated as weight in kilograms divided by squared height in meters (kg/m^2^). Participants were classified as “current”, “former”, or “never” smokers based on answers to the questions “Have you ever smoked at least 100 cigarettes in your entire life?” and “Do you NOW smoke daily, some days or not at all?”. Participants were similarly classified as “current”, “former”, or “never” drinkers based on the questions “Do you presently drink alcoholic beverages?” and “Did you ever drink alcohol?”. Depressive symptoms were measured using the Center for Epidemiological Studies Depression Scale (CESD-10), a short scale assessing symptoms of depressed affect, interpersonal relations, and positive affect^48^. Items are answered on a 4-point Likert-type scale from 0= rarely or none of the time to 3= all of the time. Total scores range from 0 to 30 with higher scores indicating more symptoms. Descriptive statistics for the full Sociocultural Ancillary Study sample (N=5,313), accounting for survey design and sampling weights, are shown in Table 1 (see supplementary Table S1 for unweighted values). The acculturation and stress measures used in these analyses were collected during the Sociocultural Ancillary Study, within 9 months (within 4 months for most) of each participant’s baseline exam^49^, well before Visit 2. Social subscale scores of the short acculturation scale for Hispanics (SASH)^50^ were incorporated in all models to control for potential health effects of social acculturative processes not defined by language use metrics. On 5-point Likert-type scales, participants rated their close friends, social gatherings, people who they visit, and their preferences for their children’s friends from 1 = All Hispanic/Latino to 5= All non-Hispanic/non-Latino, with the average of these four items taken as the participants social subscale score (range 1 to 5). Chronic stress scores ranged from 0 to 8 based on the number of stressors the participant endorsed both as occurring and lasting at least 6 months within the following areas: work, financial, relationships, personal or close person health problems, drug or alcohol problems, caregiving, or other chronic stressors. Perceived stress was measured with the 10-item Perceived Stress Scale. Participants responded on a 5-point Likert scale to measure their coping with stress during the past month. Scores range from 0 to 40 with values below 14 indicating low perceived stress and values above 26 representing high perceived stress. This measure has demonstrated high internal consistency in the English and Spanish versions in the current sample (alpha .86 in English; alpha .84 in Spanish^49^) with total computed scores for either language recommended for use in the US Hispanic/Latino population^51^. The Adverse Childhood Experiences (ACE) questionnaire has participants report whether they experienced any of 10 adverse events before age 18. These include physical, emotional, or sexual abuse, physical or emotional neglect, or household dysfunction, with scores ranging from 0 to 10. One or more stress measures were missing for 159 participants; thus, all risk factor models used a sample of 5154.

Follow-up Visit 2 interview data were used to assess chronic disease health outcomes. Prevalent disease was dichotomously coded as 0 for no diagnosis or 1 for all participants with disease present at Visit 2 (or present at Visit 1 with missing data at Visit 2). Diabetes was diagnosed using the American Diabetes Association criteria^52^. The criteria are: 1) fasting plasma glucose 126 mg/dL or greater, 2) two-hour oral glucose tolerance test glucose level 200 mg/dL or greater, 3) HbA1c level 6.5% or greater, 4) scanned or transcribed glucose-lowering medication use, and/or 5) self-report of medication use or previous diabetes diagnosis by a doctor. Coronary Heart Disease (CHD) was assessed from self-reported any previous heart attack, and cardiac procedures (angioplasty, stent, or bypass surgery) or from report that a doctor had told the participant they had a heart attack. In addition, participants who demonstrated evidence of a prior myocardial infarction from electrocardiography were classified as having CHD. Prevalence of stroke was based on self-reported medical history of previous stroke, ministroke or transient ischemic attack, or cerebrovascular procedures. Hypertension was assessed from three blood pressure readings during the clinical examination and defined as an average systolic and/or diastolic blood pressure level of at least 140/90 mm Hg or if the participant was prescribed antihypertensive medication. Chronic obstructive pulmonary disease (COPD) was self-reported by answering the prompt, “Has a doctor ever told you that you had COPD or emphysema and/or chronic bronchitis?”. From the overall sample of Sociocultural Ancillary Study participants who attended the Visit 2 examination, self-reported chronic conditions and disease outcomes had different numbers of missing items; sample sizes ranged from 4284 to 4573 participants for the chronic disease models.

### STATISTICAL ANALYSES

Analyses were conducted in R version 4.2.1 accounting for the HCHS/SOL complex survey design. Analysis code is available from the corresponding author upon request. Demographic, risk factor, and health outcome descriptive statistics were calculated across the full sample and by language preference using the *svymean, svysd, svyciprop,* and *svytable* commands of the *survey* package^53^ (Table 1). Direct comparisons between English and Spanish preferring groups accounted for survey design by utilizing the functions *svyttest* and *svychisq* (Rao & Scott adjustment of *χ*^*2*^ test) for continuous and categorical variables, respectively. For ease of interpretation, the alcohol use and smoking status categories of “former” and “current” were combined for logistic regression modeling to assess the likelihood of ever drinking alcohol or smoking as compared to “never”.

Confirmatory factor analysis was conducted to estimate a latent “general stress” variable from the three continuous stress measures (chronic stress, perceived stress, ACE) using the *cfa* function of the *lavaan* package^54^, fit using the maximum likelihood estimator with robust standard errors. These stress indicators were normally distributed (with skewness and kurtosis < 1). Factor scores were calculated for each participant, serving as the “general stress” indicator.

### ANALYTICAL MODELS

Design effects (stratification and clustering) and HCHS/SOL sample weights were accounted for in all models using the *svydesign* and associated model fit functions of the *survey* package^53^. For the associations between general stress and health risk factors, logistic regression models were fit to the binary smoking and alcohol risk factors and linear regressions to the BMI and depressive symptoms risk factors with the *svyglm* function. These models controlled for field center, age, sex, Hispanic/Latino background, highest level of education, household income, years in the US, and SASH social subscale scores. To assess the effect modification of language preference (Spanish or English), an interaction term with the latent “general stress” factor scores was included in these models. Odds ratios (and 95% confidence intervals) were estimated using comparable logistic regression models for each self-reported and clinically assessed chronic disease outcome of interest (CHD, stroke, COPD, diabetes, hypertension) incorporating the same control variables, general stress, language preference and their interaction, additionally controlling for the behavioral risk factors measured during Visit 1 (BMI, alcohol use, smoking status). When interactions terms showed *p*-values above .10, models including only main effects (i.e., excluding the interaction) were fit and are reported in the main text of the results section. When interaction terms showed *p*-values at or below .10, simple effects of stress for each language preference subgroup are reported. For ease of comparison across outcomes, fully adjusted models including interaction terms as well as stress effects for each language preference group are shown in Tables 2, 3, and 4.

## Results:

### DESCRIPTIVE ANALYSES

Table 1 shows the distribution of all sociodemographic, risk factor, and chronic disease outcome variables overall and by language preference. Persons of Mexican descent comprised the largest ethnic group (36.5%), though persons of Puerto Rican descent made up the largest proportion of those preferring English (37.7%). Most adults (78.1%) were born outside of the US and preferred Spanish (75.4%). Sociodemographic characteristics differed significantly by language preference. Specifically, Spanish preferring adults were on average older (*t*(556)=11.02, *p*<.0001), more likely to be female (*F*(1, 557)=10.23, *p*=.001, Rao & Scott adjusted *χ*^*2*^ had lower education (*F*(2, 1094)=12.54, *p*<.0001), lower incomes (*t*(554)= 6.22, *p*<.0001), were less likely to have health insurance (*F*(1, 557)= 58.28, *p*<.0001), had spent less time in the US (*t*(556)= 17.17, *p*<.0001), and tended to interact socially in more Hispanic/Latino contexts (*t*(554)= 15.22, *p*<.0001) than their English preferring counterparts. They also exhibited lower BMI (*t*(556)= 3.35, *p*=.001), were less likely to have ever smoked cigarettes (*F*(2, 1010)=24.62, *p*<.0001) or consumed alcohol (*F*(2, 1107)=26.26, *p*<.0001), and had fewer depressive symptoms (*t*(554)= 2.05, *p*=.041) than English preferring adults, before controlling for any sociodemographic factors. Subsequent models testing for stress effects on chronic disease across language preferences are thus adjusted for these sociodemographic and risk factor variables.

Participants with missing data for at least one stress variable (N=159) were, on average more likely to prefer Spanish (89.2%), older (mean ± SD, 48.5 ± 17.3 years), and less likely to be male (37.1%) than those included in the analytic sample while showing a similar distribution across Hispanic/Latino background groups (32.3% Mexican) and time spent in the US (21.3 ± 15.3 years). These participants were excluded from the risk factor and chronic disease models.

**Figure 2:**
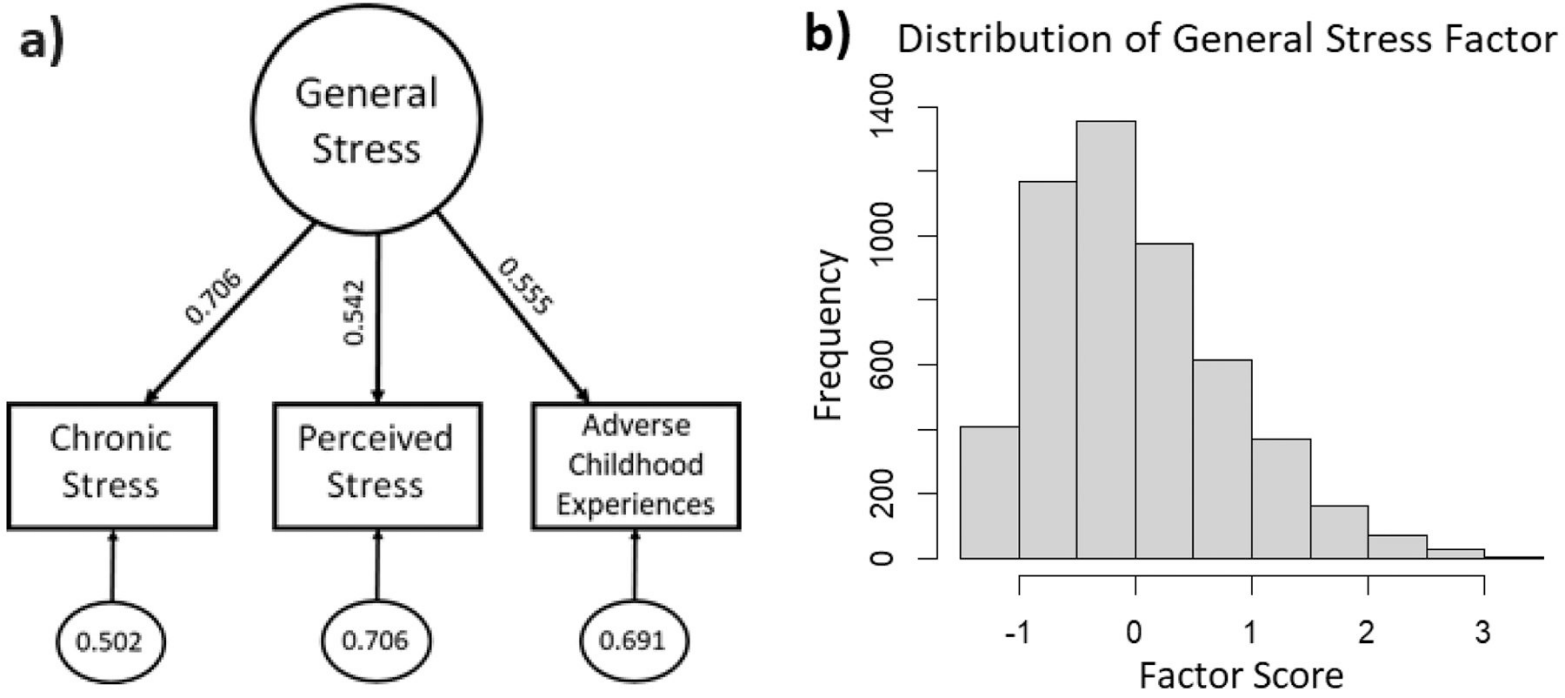
a) Factor model for latent “general stress” variable showing factor loadings and variances for the three self-report stress measures. b) distribution of latent “general stress” factor scores (N=5154)

The three stress measures were significantly lower among Spanish preferring compared to English preferring adults (chronic stress: *t*(555)= 5.48; perceived stress: *t*(556)= 4.79; ACE: *t*(556)= 6.08; all *p*’s<.0001). These measures further showed significant positive bivariate correlations (chronic and perceived stress: *r*= .383; chronic stress and ACE: *r*= .391; perceived stress and ACE: *r*= .303; all *p*’s<.0001), thus resulting in significant positive loadings of similar magnitude (0.706, 0.542, 0.555 for chronic stress, perceived stress, and ACE, respectively) in the just identified “general stress” model (Figure 2a). This latent factor exhibited a slight positive skew (Figure 2b), likely due to floor effects of the self-report measures used in its calculation (i.e., participants could not report negative numbers of chronic or childhood stressors). As with the observed stress indicators, factor scores for general stress were significantly higher among English preferring persons (0.270 ± 0.883) compared to those preferring Spanish (−0.065 ± 0.773; *t*(1386)= 11.01, *p*<.0001).

Before controlling for sociodemographic variables or behavioral risk factors, diabetes (38.8% vs 30.5%) and hypertension (26.4% vs 38.8%) showed the largest differences in prevalence across language preferences. Differences across language preferences were smaller for stroke, COPD, and CHD. However, relative prevalence across groups must be interpreted in light of models incorporating confounding effects of variables such as age and BMI (see below) which could explain differences appearing between English and Spanish preferring groups.

### ASSOCIATION OF STRESS AND LANGUAGE PREFERENCE WITH BEHAVIORAL FACTORS AND DEPRESSIVE SYMPTOMS

After adjusting for sociodemographic variables, we saw evidence for language by stress interactions on alcohol use and weaker associations with BMI (Table 2), such that increases in stress were associated with a higher likelihood of drinking alcohol (interaction OR= 1.61, *p*=.038) and higher BMI (interaction B=0.91, *p*=.100) for persons preferring English relative to Spanish. That is, a one-point rise in general stress corresponded to 1.33 kg/m^2^ (95% CI [0.34, 2.32]) higher BMI for those preferring English but only 0.42 kg/m^2^ (95% CI [0.08, 0.75]) higher BMI for those preferring Spanish. Similarly, each unit rise in general stress was associated with nearly doubled odds of alcohol use in English preferring persons (OR= 1.99 [1.29, 3.06]) and a more modest 23% increase among those preferring Spanish (OR= 1.23 [1.06, 1.45]). In contrast, smoking status and depressive symptoms showed no evidence for stress by language preference interactions. When excluding interaction effects (models not shown), a one unit increase in the general stress factor was associated with 37% increased odds of smoking (OR=1.37, 95% CI [1.21, 1.56], *p*<.0001) and a 2.89 point rise in CES-D measured depressive symptoms (B=2.89 [2.56, 3.22], *p*<.0001; 0 to 30 scale, scores above 10 considered depressed^48^). Moreover, English language preference was associated with 97% increased odds of smoking, when controlling for stress and sociodemographic variables (OR=1.97 [1.45, 2.69], *p*<.0001). Therefore, relative to those preferring English, Spanish preferring Hispanic/Latino adults appear to be partially protected from the negative consequences of stress on these behavioral health risk factors, independent of other acculturative variables like ethnic background, social relationships, and time in the US (see supplementary Table S2 for odds ratios of control variables).

### ASSOCIATION OF STRESS AND LANGUAGE PREFERENCE WITH CHRONIC DISEASE PREVALENCE

**Figure 3:**
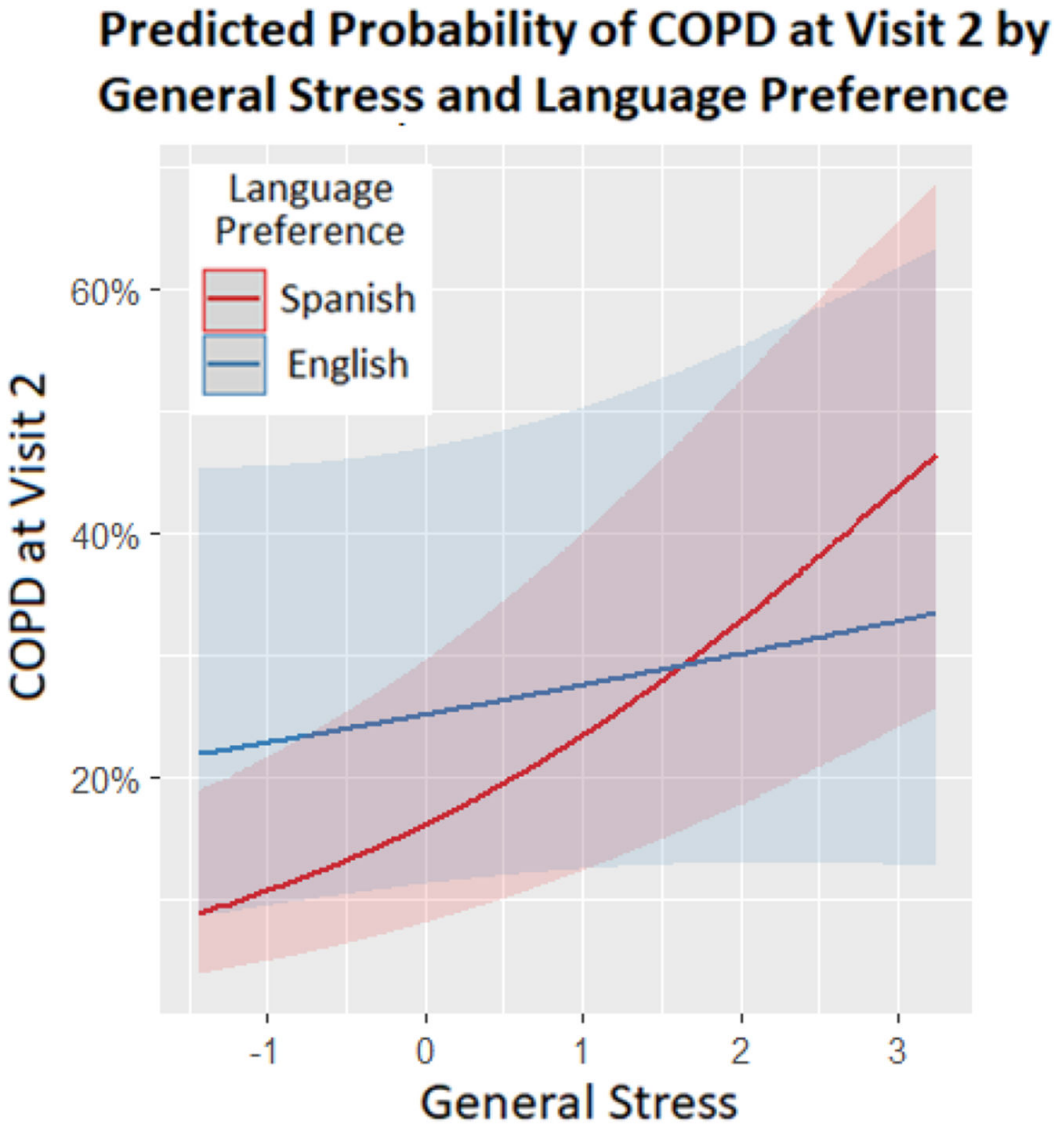
Plotted relationship between predicted likelihood of COPD vs General Stress by Language Preference. Error bands represent 95% confidence intervals.

In the fully adjusted models (controlling for sociodemographic and behavioral risk factors), only self-reported COPD prevalence showed a significant moderation of general stress effects by language preference (interaction OR= 0.70, *p*=.020; Figure 3). Language preference stratified analyses showed that a one unit increase in general stress was associated with 61% increased odds of reporting COPD for Spanish preferring persons (OR= 1.61 [1.32, 1.96]), whereas this relationship was not significant among those interviewed in English (OR=1.13 [0.85, 1.48]). This stronger stress effect meant that Spanish preferring persons with low general stress (below ~+1.5, most individuals) showed lower self-reported COPD prevalence than English preferring persons. Controlling for general stress (excluding the interaction), preference for English was associated with 62% increased odds of reported COPD (OR=1.62 [0.98, 2.68], *p*=.061) compared to Spanish preference. While not varying significantly across language preferences, a unit increase in general stress was also associated with 41% increased odds of self-reported CHD (OR= 1.41 [1.14, 1.73], *p*=.001) and marginally associated with 15% increased odds of diabetes (OR= 1.15 [1.00, 1.31], *p*=.051) when excluding interaction effects (models not shown). Adjusting for covariates, CHD prevalence (OR=1.27 [0.66, 2.42]) was slightly higher and diabetes prevalence (OR=0.81 [0.54, 1.22]) slightly lower among English compared to Spanish preferring persons, though not significantly so (models not shown). Regression coefficients and relevant odds ratios for these full models, including interaction terms and sociodemographic and behavioral risk factors (see Table S3), are reported in Tables 3 and 4. Neither self-reported stroke (Table 3) nor clinically assessed hypertension (Table 4) were significantly predicted by general stress, language preference or their interaction in the fully adjusted models. When excluding interaction effects, a main effect of stress on hypertension emerged such that one unit increase in general stress predicted 16% greater odds of hypertension (OR= 1.16 [1.00, 1.35], *p*=.046; model not shown). After controlling for behavioral risk factors and stress effects, hypertension prevalence was slightly lower and self-reported stroke prevalence slightly higher for persons who preferred English relative to Spanish (OR=0.83 [0.56, 1.21]; OR=1.73 [0.67, 4.51]; respectively), though neither outcome varied significantly by language preference. In total, greater general stress related to higher prevalence of diabetes, hypertension, and self-reported CHD and COPD, while stress effects on COPD prevalence were dampened among those who preferred English relative to Spanish preferring adults. Controlling for sociodemographic and behavioral risk factors, COPD showed higher overall prevalence among those preferring English, while the remaining chronic diseases did not vary significantly by language preference.

## Discussion:

This investigation offers insight into the role of language preference (English vs Spanish) in differential stress effects on behavioral and biological health outcomes among diverse Hispanic/Latino adults using data from the HCHS/SOL Sociocultural Ancillary Study. As the need to look beyond simple pan-ethnic groupings in describing risk factor, respiratory disease, and CVD prevalence has been noted previously^49^, these results highlight the health and sociocultural salience of language. By combining three relevant self-reported stress indicators (chronic stress, perceived stress, adverse childhood experiences [ACE]), these models exhibited stronger relationships to the CVD related health indicators of interest (diabetes: 1.15, CHD: 1.41, COPD, 1.61) than single stress variables tested previously (diabetes: 1.20, CHD: 1.22)^1^ (diabetes: 0.98, CHD: 1.08, COPD: 1.07)^13^. Overall, greater general stress was associated with higher BMI, depressive symptoms, and likelihood of smoking and alcohol use at baseline, with elevated likelihood of smoking among English preferring persons. We further found evidence for stronger associations between stress and alcohol use among English preferring Hispanic/Latino adults. Moreover, higher general stress was associated with increased prevalence of diabetes, hypertension, and self-reported CHD and COPD; stress effects on COPD prevalence were moderated by language preference, being stronger among those preferring Spanish. These language preference effects emerged even when controlling for other acculturative variables such as time in the US, nativity, social acculturation, and Hispanic/Latino background. These findings therefore emphasize how language preference may align with differences in reactivity to a wide breadth of stress experiences, affecting the prevalence of respiratory disease and CVD related outcomes. Our understanding of the paradoxical relationship between various health risk factors and disease prevalence among Hispanic/Latino adults in the US may benefit from more consistent consideration of language and related sociocultural factors.

Higher self-reported COPD prevalence associated with increased general stress exhibited a noteworthy moderation by language preference, with this positive relationship being significantly stronger among Spanish (compared to English) preferring Hispanics/Latinos. This finding runs counter to our hypothesis of lower stress reactivity fostered by the Spanish language. Though stress had a larger impact for them, Spanish preferring participants still showed lower self-reported COPD prevalence than English preferring participants overall, in line with patterns of health outcomes across language preferences observed previously^35,36^. That is, most of the sample produced relatively low general stress scores (−1 to +1, standardized measure), tied to low COPD prevalence. This result is similar to those of Barr et al.^55^ who report significantly higher COPD prevalence among Puerto Rican-background persons, the group with the highest English preference, in the full HCHS/SOL. This model controlled for income, education, and health insurance status, indicating that these effects are not simply due to lower socioeconomic status among Spanish preferring Hispanics/Latinos. Given that smoking is a primary risk factor for COPD^56^, one could easily attribute this pattern to the significantly higher smoking rates associated with English preference in both the present sample and a previous sample of midwestern Hispanic/Latino farm workers^57^. However, this model controlled for smoking status in the sample, suggesting that aspects of the Spanish language itself could influence the stress to COPD relationship above and beyond alterations in smoking behavior. The more robust emotional lexicon noted of the Spanish language^31^ and grammatically ingrained means of minimizing (or exaggerating) experiences could serve a protective function for Hispanic/Latino individuals processing low to moderate levels of stress while accentuating or deepening the health impact of stressful experiences among those facing the highest levels of stress. Such a mechanism would corroborate models of cultural influences on stress appraisals and downstream health consequences^24^, though this interpretation must be validated through further observational research and experimental manipulations. Alternatively, linguistic differences in how events are described and expressed in English and Spanish could contribute to this pattern at the level of stress report. Indeed, the checklist approach to measuring stress (e.g., ACE) is noted to be limited by variation in individuals’ interpretations of what constitutes a “stressful” event^1^. This perspective is reinforced by overall higher stress measures among English preferring persons and could be addressed in models testing for mediation of language effects by stress report.

Our models additionally corroborate prior research showing consistent connections between stress and behavioral health measures^13,58-60^ while further suggesting these CVD risk factors may vary meaningfully across English- and Spanish-preferring Hispanic/Latino individuals. Firstly, the stronger stress to alcohol use relationship seen among English-preferring persons could indicate shifts in the social norms and perceptions predictive of drinking behavior, mirroring patterns noted between Hispanic/Latino and non-Hispanic White university students^61,62^. While high familial and social support may buffer against stress effects on health behaviors,^63,64^ this result suggests that emotional processing in the Spanish language may further help people cope with stressors without turning to alcohol. The significant positive association between BMI and general stress supports earlier work linking chronic and perceived stress to diet quality and obesity prevalence^59^ and ACE report to BMI^13^. Moreover, persons who preferred English exhibited higher BMI and a slightly stronger stress-BMI association than those preferring Spanish. This pattern reflects that displayed between foreign- and US-born Hispanics/Latinos, as multi-domain cumulative stress can explain cross-ethnic obesity differences between non-Hispanic Whites and US-born (but not immigrant) Hispanic/Latino adults^65^. Although US-born persons in this sample were far more likely to prefer English, these language preference effects remain even after controlling for both nativity and time in the US (models not shown). This result may help explain why greater language discordance between Hispanic/Latino parents and children (higher child English preference) is linked to increased risk of childhood obesity^66^. As stress responses have long been associated with smoking and drinking behaviors in the general population^64^, these findings build upon work linking stressors like racial/ethnic discrimination to smoking, drinking, and binge drinking rates among Hispanic/Latino youth and adults^58,60^. Higher smoking rates among non-Hispanic whites as compared to non-migrant, migrant, and US-born Hispanic/Latino populations^67^ could contribute to increased smoking prevalence in the English preference group, as those persons reported more social interactions in non-Hispanic/non-Latino contexts than the Spanish-preferring group. However, differences in social acculturation were controlled for (via SASH social subscale), suggesting additional mechanisms related to language use are at play. In total, while Hispanics/Latinos’ general stress shows a consistent association with the expression of behavioral health risk factors, these health indicators also show noteworthy variation according to language preference.

This investigation observed a strong relationship between stress and depressive symptoms, a key mental health risk factor, with general stress presenting the largest effect size of all tested variables. These findings mirror previous work showing that life adversity stresses relate to depressive symptoms within the HCHS/SOL sample^68,69^ and that acculturative stress strongly predicts depression report in Hispanic/Latino youth^70^. Thus, more comprehensive approaches to the stress experience, especially those which function similarly across Hispanic/Latino groups^71^, may better predict depressive symptoms than singular measures like the Hispanic Stress Inventory (HIS2) which did not significantly relate to depression report in a diverse sample of Hispanic/Latino immigrants^72^. The strong positive relationship between stress and depression was consistent across language preferences.

These analyses likewise substantiate previously established connections between life stress (particularly chronic stress) and incidence and prevalence of CHD^73^, hypertension^1^, and diabetes^74^ (see for a review^75^). The lack of moderation by language preference parallels the significant relationship between chronic stress and metabolic syndrome found among Mexican and Puerto Rican Americans, which remains consistent across levels of social support^76^. The inverse relationships reported by Gallo, Roesch, et al.^1^ with specific stress measures and CVD outcomes may explain the relatively weak stress effect for diabetes and hypertension, as chronic stress and traumatic event effects of similar magnitudes but opposite directions may partially cancel out. Conversely, positive general stress associations with CHD may have emerged due to larger positive chronic stress effects relative to weaker effects of traumatic life events. This composite “general stress” indicator therefore represents a meaningful contributor to prevalence patterns of several CVD related chronic diseases among the US Hispanic/Latino population.

Overall, this investigation emphasizes stress as a key health indicator among the Hispanic/Latino population, while further suggesting that Spanish language use may play a role in stress appraisal and processing, potentially contributing to the Hispanic paradox via effects on both risk factors and chronic disease. This study joins a growing body of literature linking Spanish language preference with health protective factors such as medication adherence^77^, improved sleep quality compared to non-Hispanic Whites^78^, and better patterns of surgical intervention for colorectal cancer^79^ among Hispanic/Latino patients. These patterns could be partially related to greater expression of Hispanic/Latino cultural values, such as familism, shown to buffer against negative health consequences of stress and risky health behaviors^80,81^, though importantly they emerge even controlling for participants’ social acculturation (i.e., relative interaction with Hispanic/Latino vs non-Hispanic/non-Latino contexts). As differential exposure and reactivity to stress is known to relate to health disparities in disorder trajectory and treatment response across populations^82^, the role of language use during stress appraisal, above and beyond general acculturative processes, requires additional scrutiny. Whether being primarily driven by specific features of Spanish or cultural values and practices tied to its use, the present analyses and earlier studies suggest language preference could meaningfully contribute to our knowledge of the health resiliency of the US Hispanic/Latino population.

Relevant limitations of the study sample and analytic approach must be considered when interpreting these results and shaping future investigations. To begin, low total prevalence of CHD and stroke in this population weakened our ability to detect potential relationships between our variables of interest and these outcomes. Additionally, self-reported disease, such as the CHD, stroke, and COPD outcomes used here, is an imperfect indicator of objective disease, as low specificity has been noted between self-reported disease and objective measures of heart failure and COPD^83,84^. However, some research suggests self-report sensitivity is better for estimates of prevalence as compared to incidence^43,44^. Moreover, the risk factor models are cross-sectional, while longitudinal models for the health outcomes included participants with disease at either Visit 1 or 2. It remains to be seen whether the associations described here remain consistent, strengthen, or dissipate over a longer timeframe. We thus caution against strong interpretation before these results are independently replicated. While we found evidence for variation in stress effects across language preferences, we incorporated neither measures of proficiency in either language nor metrics of bilingual competence. Therefore, we cannot conclude that these patterns would hold for continuous measures of language usage as opposed to our dichotomized preference variable. Furthermore, while our composite general stress variable showed a stronger association to our tested health outcomes than single indicators in the past, this metric did not incorporate culturally sensitive stress measures, such as discrimination, acculturative stress, or specific stressors related to the immigration experience, which may likely be more sensitive to language preference effects. Moreover, the present analyses cannot determine whether observed differences in prevalence rates and stress effects across language preference groups should be ascribed to aspects of the English and Spanish languages themselves or whether language use correlates with other cultural values or acculturative processes (outside of social interactions) which serve health protective functions.

Observational designs with a broader range of acculturative and language use (e.g., proficiency, competence, accent) variables or experimental studies investigating the relationship between language and stress appraisal or reactivity would help to clarify these results. Finally, it is possible that rather than the strength of the stress to health relationship differing across languages, the language in which an event is experienced or processed influences the degree to which it is internalized as stressful, thus altering downstream impacts for health; this possibility could be examined through a formal mediation analysis. These limitations should be considered in future studies to build upon and clarify the findings presented here.

## Conclusion

In sum, this study found consistent evidence for a link between general stress and important health risk factors and respiratory and CVD related chronic diseases in a diverse cohort of Hispanic/Latino adults. Specifically, greater stress was associated with increased likelihood to smoke and drink alcohol, elevated BMI and depressive symptoms, and higher prevalence of diabetes, hypertension, and self-reported CHD and COPD. We further found that smoking rates were higher and the stress to alcohol use (and to a lesser extent BMI) relationships were stronger for English preferring Hispanic/Latino adults relative to those who prefer Spanish. Spanish preference was associated with a stronger stress to COPD prevalence relationship, though English preferring persons exhibited higher COPD prevalence overall. These findings can be developed upon to elucidate the role of language in the perception, appraisal, and ultimate health consequences of stress, potentially serving to improve treatment outcomes and reduce health disparities across a wide diversity of populations.

## Supplementary Material

1

## Figures and Tables

**Table 1:** Weighted Demographic and Outcome Variables: HCHS/SOL Sociocultural Ancillary Study

	Total	English Preference	Spanish Preference
**N**	**5313**	**1017**	**4296**
Sex*, **% male**	45.1%	51.7%	43.0%
Health insurance*, **% insured**	52.3%	69.6%	46.7%
Age*, **mean (SD) years**	42.5 (15.0)	35.0 (14.3)	44.9 (14.5)
18-44	56.5%	74.2%	50.7%
45-64	33.6%	21.3%	37.6%
65-74	9.9%	4.4%	11.7%
**Hispanic/Latino Heritage Group** * **, %**			
Central American	12.4%	5.0%	14.8%
Cuban	20.3%	5.7%	25.1%
Dominican	11.7%	11.1%	11.9%
Mexican	36.5%	31.5%	38.2%
Puerto Rican	15.8%	37.7%	8.6%
South American	3.3%	9.0%	1.5%
**Education** * **, %**			
> High school or GED	32.5%	23.3%	35.5%
High school or GED	28.0%	29.4%	27.6%
< High school or GED	39.4%	47.3%	36.9%
**Household Income** * **, %**			
< $10,000	16.2%	12.5%	17.3%
$10,000-20,000	30.7%	24.8%	32.6%
$20,001-40,000	28.9%	29.0%	28.9%
$40,001-75,000	10.9%	18.5%	8.5%
> $75,000	4.6%	7.9%	3.5%
Not Reported	8.7%	7.2%	9.3%
**Smoking Status** * **, %**			
Never	61.3%	52.5%	64.1%
Former	1 8.0%	15.3%	19.0%
Current	20.7%	32.2%	16.9%
**Alcohol Use** * **, %**			
Never	20.0%	9.3%	23.4%
Former	30.4%	28.8%	31.0%
Current	49.6%	61.9%	45.6%
Years living in US*, **mean (SD) years**	20.5 (14.8)	32.1 (14.2)	16.8 (13.0)
Born in the US	21.9%	66.5%	7.4%
< 2 Years	8.2%	0.5%	10.7%
3-5 Years	8.5%	0.7%	11.0%
6-10 Years	14.7%	2.1%	18.9%
11-15 Years	11.6%	2.8%	14.5%
>15 Years	35.0%	27.4%	37.5%
BMI*, **mean (SD) kg/m^2^**	29.6 (6.3)	30.8 (7.4)	29.3 (5.8)
<25 kg/m^2^	22.1%	21.8%	22.2%
25-29.9 kg/m^2^	36.9%	28.9%	39.5%
30-34.9 kg/m^2^	24.6%	25.0%	24.5%
≥35 kg/m^2^	16.4%	24.2%	13.9%
SASH Social Subscale*, **mean (SD)**	2.24 (0.6)	2.64 (0.6)	2.11 (0.6)
Depressive Symptoms*, **mean (SD)**	7.3 (6.2)	7.8 (6.4)	7.1 (6.1)
**Stress, mean (SD)**			
Chronic Stress*	1.8 (1.6)	2.2 (1.8)	1.7 (1.5)
Adverse Childhood Experiences*	2.50 (2.3)	3.1 (2.4)	2.3 (2.3)
Perceived Stress*	14.9 (6.8)	16.2 (6.8)	14.4 (6.7)
**Diabetes at Visit 2, %** ^a^	27.9%	38.8%	30.5%
**Hypertension at Visit 2, %** ^a^	36.0%	26.4%	38.8%
**CHD at Visit 2, %** ^a^	5.1%	5.9%	4.8%
**Stroke at Visit 2, %** ^a^	1.9%	1.6%	1.9%
**COPD at Visit 2, %** ^a^	12.0%	15.8%	10.9%

Note: N is unweighted, means and percentages are weighted. *Variables showing significant (*p*<.05) variation across language preference subgroups based on *t*-tests for continuous variables and χ^2^ test for categorical variables. ^a^These percentages reflect proportions excluding participants with missing values for the given health outcome.

**Table 2:** Linear and Logistic Regression Analyses examining the Interaction between Language Preference and Stress on Health Risk Factors

	Alcohol Use^a^		Cigarette Smoking^a^		BMI		Depressive Symptoms	
	Beta	Standard Error	Beta	Standard Error	Beta	Standard Error	Beta	Standard Error
General Stress	*0.213	0.080	*0.241	0.072	*0.416	0.172	*2.867	0.207
Language Preference^b^	0.110	0.234	*0.642	0.149	*0.967	0.483	0.021	0.325
General Stress by Language Preference^b^	*0.474	0.228	0.262	0.192	†0.913	0.560	0.082	0.321
General Stress by Language Preference	Adjusted Odds Ratio [95% CI]		Adjusted Odds Ratio [95% CI]		Beta [95% CI]		Beta [95% CI]	
**Spanish**	*1.23 [1.06, 1.45]		*1.27 [1.10, 1.47]		*0.42 [0.08, 0.75]		*2.87 [2.46, 3.27]	
**English**	*1.99 [1.29, 3.06]		*1.65 [1.19, 2.31]		*1.33 [0.34, 2.32]		*2.95 [2.45, 3.45]	

Note: N=5,154; odds ratios and slopes are adjusted for age, sex, study site, Hispanic/Latino background, education, health insurance status, income, years in the US, SASH social subscale. ^a^Dichotomized as current or former smoking/drinking vs. never smoking/drinking. ^b^Coded as 0-Spanish, 1-English; results reflect English preference effects. *p<.05, †*p*<.10

**Table 3:** Logistic Regression Analyses examining Interaction between Language Preference and General Stress on Self-Reported Chronic Disease Outcomes

	CHD		Stroke		COPD	
**N**	4325		4284		4324	
	Beta	Standard Error	Beta	Standard Error	Beta	Standard Error
General Stress	*0.335	0.120	0.201	0.205	*0.477	0.100
Language Preference^a^	0.230	0.347	0.426	0.523	*0.603	0.255
General Stress by Language Preference^a^	0.019	0.261	0.253	0.366	*−0.360	0.154
General Stress by Language Preference	Adjusted Odds Ratio [95% CI]		Adjusted Odds Ratio [95% CI]		Adjusted Odds Ratio [95% CI]	
**Spanish**	*1.40 [1.1 0, 1.77]		1.22 [0.82, 1.83]		*1.61 [1.32, 1.96]	
**English**	1.42 [0.92, 2.20]		1.57 [0.87, 2.85]		1.13 [0.85, 1.48]	

Note: Odds ratios are adjusted for age, sex, study site, Hispanic/Latino background, education, health insurance status, income, years in the US, SASH social subscale, smoking status, alcohol use, BMI. ^a^Coded as 0-Spanish, 1-English; results reflect English preference effects. *p<.05

**Table 4:** Logistic Regression Analyses examining Interaction between Language Preference and General Stress on Clinically Assessed Chronic Disease Outcomes

	Hypertension		Diabetes	
**N**	4573		4481	
	Beta	Standard Error	Beta	Standard Error
General Stress	0.087	0.080	*0.187	0.080
Language Preference^a^	−0.243	0.210	−0.173	0.210
General Stress by Language Preference	0.238	0.190	−0.183	0.148
General Stress by Language Preference^a^	Adjusted Odds Ratio [95% CI]		Adjusted Odds Ratio [95% CI]	
**Spanish**	1.09 [0.93, 1.28]		*1.21 [1.03, 1.41]	
**English**	1.38 [0.99, 1.94]		1.00 [0.78, 1.29]	

Note: Odds ratios are adjusted for age, sex, study site, Hispanic/Latino background, education, health insurance status, income, years in the US, SASH social subscale, smoking status, alcohol use, BMI. ^a^Coded as 0-Spanish, 1-English; results reflect English preference effects. *p<.05
