# Supplementary material for: Language Preference and its Moderating Role in Coping with Stress: The Hispanic Community Health Study/Study of Latinos
Source: Med Res Arch. Author manuscript; Available in PMC 2024 Jun 6. (PMC11156427; doi:10.18103/mra.v11i10.4625)
Supplement: 1 [file NIHMS1945573-supplement-1.pdf]

Supplementary Material

Table S1: Unweighted demographic and outcome variables: the HCHS/SOL Sociocultural Ancillary Study

	Total	Preference for English	Preference for Spanish
**N**	5313	1017	4296
**Sex*, N (%) male**	2014 (37.9%)	456 (44.8%)	1558 (36.3%)
**Health Insurance*, N (%)**	2670 (50.3%)	708 (69.6%)	1962 (45.7%)
**Age*, mean (SD) years**	46.64 (13.65)	39.10 (14.58)	48.42 (12.78)
18-44	2035	595	1440
45-64	2825	388	2437
65-74	453	34	419
**Hispanic/Latino Heritage Group*, N (%)**			
Dominican	534 (10.1%)	74 (7.3%)	460 (10.7%)
Central American	903 (17.0%)	59 (5.8%)	844 (19.6%)
Cuban	775 (14.6%)	43 (4.2%)	732 (17.0%)
Mexican	2080 (39.2%)	315 (31.0%)	1765 (41.1%)
Puerto Rican	880 (16.6%)	443 (43.6%)	437 (10.2%)
South American	137 (2.6%)	83 (8.2%)	54 (1.3%)
**Education*, N (%)**			
No high school diploma or GED	1923 (36.2%)	243 (23.9%)	1680 (39.1%)
At most high school diploma or GED	1383 (26.0%)	282 (27.7%)	1101 (25.6%)
More than high school diploma or GED	1998 (37.6%)	492 (48.4%)	1506 (35.1%)
**Household Income*, N (%)**			
< $10,000	888 (16.7%)	144 (14.2%)	744 (17.3%)
$10,000-20,000	1673 (31.5%)	244 (24.0%)	1429 (33.3%)
$20,001-40,000	1577 (29.7%)	292 (28.7%)	1285 (29.9%)
$40,001-75,000	556 (10.5%)	192 (18.9%)	364 (8.5%)
> $75,000	178 (3.4%)	80 (7.9%)	98 (2.3%)
Not Reported	441 (8.3%)	65 (6.4%)	376 (8.8%)
**Years living in US*, mean (SD)**	22.5 (15.4)	35.6 (14.7)	19.4 (13.8)
Born in the US, N (%)	917 (17.3%)	671 (66.0%)	246 (5.7%)
< 2 years, N (%)	364 (6.9%)	5 (0.5%)	359 (8.4%)
3-5 years, N (%)	390 (7.3%)	6 (0.6%)	384 (8.9%)
6-10 years, N (%)	728 (13.7%)	19 (1.9%)	709 (16.5%)
11-15 years, N (%)	571 (10.7%)	24 (2.4%)	547 (12.7%)
>15 years, N (%)	2340 (44.0%)	292 (28.7%)	2048 (47.67%)
**BMI*, mean (SD) kg/m²**	29.9 (6.2)	30.7 (7.3)	29.8 (5.9)
<25 kg/m², N (%)	1047 (19.7%)	219 (21.6%)	828 (19.3%)
25-29.9 kg/m², N (%)	1961 (36.9%)	298 (29.3%)	1663 (38.7%)
30-34.9 kg/m², N (%)	1385 (26.1%)	260 (25.6%)	1125 (26.2%)
≥35 kg/m², N (%)	909 (17.1%)	236 (23.2%)	673 (15.7%)
**Smoking Status*, N (%)**			
Never	3240 (61.0%)	506 (49.8%)	2734 (63.6%)
Former	1094 (20.6%)	200 (19.7%)	894 (20.8%)
Current	974 (18.3%)	311 (30.6%)	663 (15.4%)
**Alcohol Use*, N (%)**			
Never	1105 (20.8%)	98 (9.6%)	1007 (23.4%)
Former	1752 (33.0%)	336 (33.0%)	1416 (33.0%)
Current	2454 (46.2%)	581 (57.1%)	1873 (43.6%)
**SASH Social Subscale*, mean (SD)**	2.19 (0.6)	2.62 (0.5)	2.09 (0.6)
**Depressive Symptoms*, mean (SD)**	7.51 (6.29)	8.15 (6.44)	7.36 (6.24)
**Stress, mean (SD)**			
Chronic Stress*	1.88 (1.66)	2.35 (1.87)	1.77 (1.58)
Adverse Childhood Experiences*	2.48 (2.34)	3.15 (2.45)	2.32 (2.28)
Perceived Stress*	14.81 (6.85)	16.14 (7.07)	14.49 (6.76)
**Diabetes at Visit 2, N (%)[a]**	1697 (37.9%)	269 (34.3%)	1428 (38.6%)
N/A	832 (15.7%)	232 (22.8%)	600 (14.0%)
**Hypertension at Visit 2, N (%)[a]**	2238 (48.9%)	336 (41.6%)	1902 (50.5%)
N/A	740 (13.9%)	210 (20.6%)	530 (12.3%)
**CHD at Visit 2, N (%)[a]**	340 (7.9%)	56 (7.4%)	284 (8.0%)
N/A	988 (18.6%)	258 (25.4%)	730 (16.7%)
**Stroke at Visit 2, N (%)[a]**	105 (2.5%)	23 (3.0%)	82 (2.3%)
N/A	1029 (19.4%)	260 (25.6%)	769 (17.9%)
**COPD at Visit 2, N (%)[a]**	629 (14.5%)	176 (22.9%)	453 (12.7%)
N/A	989 (18.6%)	249 (24.5%)	740 (17.2%)

*Variables showing significant (p<.05) variation across language preference subgroups based on t-tests for continuous variables and χ^2 test for categorical variables. [a]These percentages reflect proportions excluding participants with missing values for the given outcome.

Table S2: Association between Control Variables and Behavioral and Mental Health Risk Factors at Baseline: HCHS/SOL Sociocultural Ancillary Study

	Odds Ratio [95% Confidence Interval]		Beta [95% Confidence Interval]	
	**Alcohol**[a]	**Smoking**[a]	**BMI**	**Depressive Symptoms**
**Age**	*0.99 [0.98, 1.00]	*1.02 [1.01, 1.03]	*0.04 [0.02, 0.06]	0.00 [–0.02, 0.02]
**Sex**[b]	*3.22 [2.55, 4.07]	*2.59 [2.10, 3.18]	*–1.30 [–1.80, –0.79]	*–1.32 [–1.86, –0.78]
**Hispanic/Latino Heritage Group**				
Dominican	1.60 [0.76, 3.14]	0.89 [0.54, 1.46]	0.54 [–1.10, 2.17]	0.77 [–0.65, 2.19]
Central American	1.02 [0.57, 1.85]	0.92 [0.61, 1.38]	0.11 [–0.89, 1.11]	*1.03 [0.15, 1.90]
Cuban	0.97 [0.50, 1.90]	1.38 [0.87, 2.19]	–0.30 [–1.81, 1.21]	*2.16 [1.14, 3.19]
Mexican[c]	1.00	1.00	——	——
Puerto Rican	1.03 [0.55, 1.93]	1.44 [0.99, 2.12]	0.97 [–0.13, 2.07]	*1.86 [0.90, 2.82]
South American	0.94 [0.28, 3.15]	*3.07 [1.65, 5.70]	0.04 [–1.64, 1.71]	0.66 [–1.36, 2.67]
**Years in the U.S.**	1.01 [1.00, 1.02]	1.00 [0.99, 1.01]	*0.04 [0.02, 0.06]	*0.02 [0.00, 0.05]
**Education**				
No high school diploma or GED[c]	1.00	1.00	——	——
High school diploma or GED	0.82 [0.63, 1.07]	0.79 [0.62, 1.00]	–0.61 [–1.31, 0.09]	–0.81 [–1.73, 0.11]
> high school diploma or GED	1.03 [0.80, 1.34]	*0.66 [0.52, 0.83]	*– 1.02 [–1.65, –0.38]	*–1.07 [–1.92, –0.21]
**Income**	1.05 [1.00, 1.11]	*0.92 [0.88, 0.96]	0.01 [–0.10, 0.12]	*–0.41 [–0.55, –0.28]
**Field center**				
Bronx, NY[c]	1.00	1.00	——	——
Chicago, IL	0.73 [0.43, 1.23]	1.11 [0.81, 1.53]	–0.19 [–1.08, 0.70]	0.18 [–0.66, 1.03]
Miami, FL	*0.21 [0.14, 0.33]	1.30 [0.90, 1.89]	0.39 [–1.00, 1.78]	–0.61 [–1.49, 0.27]
San Diego, CA	1.00 [0.49, 2.02]	1.27 [0.87, 1.86]	–0.60 [–1.64, 0.49]	0.47 [–0.54, 1.48]
**Health Insurance Status**[d]	0.94 [0.73, 1.20]	0.96 [0.76, 1.20]	*–0.69 [–1.30, –0.08]	–0.23 [–0.96, 0.51]
**SASH Social Subscale**	0.95 [0.76, 1.18]	0.91 [0.75, 1.09]	0.27 [0.08, 0.75]	–0.09 [–0.71, 0.53]

Note: N=5,154. [a]Dichotomized as current or former smoking/drinking vs never smoking/drinking. [b]Female as reference group; odds ratios and slopes represent effect of being male. [c]Categories with odds ratios of 1.00 or no recorded slope reflect the reference group for each variable. [d]No health insurance as reference group; odd ratios and slopes represent effect of having health insurance. *p<.05

Table S3: Associations between Control Variables and Chronic Health Outcomes: HCHS/SOL Sociocultural Ancillary Study

	Odds Ratio [95% Confidence Interval]				
	**CHD**	**Stroke**	**COPD**	**Hypertension**	**Diabetes**
**N**	4325	4284	4324	4573	4481
**Age**	*1.07 [1.05, 1.09]	*1.08 [1.05, 1.11]	*1.04 [1.02, 1.05]	*1.10 [1.09, 1.12]	*1.06 [1.05, 1.07]
**Sex**[a]	1.45 [0.95, 2.22]	1.12 [0.55, 2.27]	*0.58 [0.41, 0.83]	*1.60 [1.25, 2.06]	*1.47 [1.13, 1.90]
**Hispanic background**					
Dominican	0.63 [0.26, 1.53]	1.36 [0.44, 4.20]	1.17 [0.54, 2.52]	1.50 [0.84, 2.70]	1.03 [0.55, 1.93]
Central American	0.51 [0.19, 1.35]	0.87 [0.30, 2.47]	1.21 [0.62, 2.36]	0.88 [0.51, 1.50]	0.81 [0.47, 1.40]
Cuban	0.47 [0.16, 1.41]	0.51 [0.14, 1.79]	1.96 [0.92, 4.19]	1.46 [0.72, 2.96]	0.75 [0.39, 1.42]
Mexican[b]	1.00	1.00	1.00	1.00	1.00
Puerto Rican	0.64 [0.28, 1.46]	0.91 [0.35, 2.36]	*3.20 [1.80, 5.68]	1.19 [0.72, 1.97]	1.49 [0.72, 3.10]
South American	1.39 [0.26, 7.45]	2.71 [0.60, 12.29]	*2.76 [1.15, 6.67]	0.89 [0.29, 2.00]	1.55 [0.66, 3.63]
**Years in the U.S.**	1.01 [0.99, 1.03]	1.01 [0.99, 1.04]	0.99 [0.98, 1.00]	1.00 [0.99, 1.01]	0.99 [0.98, 1.01]
**Education**					
No high school diploma or GED[b]	1.00	1.00	1.00	1.00	1.00
High school diploma or GED	0.73 [0.44, 1.20]	0.90 [0.37, 2.22]	0.93 [0.63, 1.37]	0.84 [0.62, 1.14]	0.77 [0.59, 1.02]
> high school diploma or GED	0.94 [0.64, 1.40]	1.23 [0.54, 2.83]	0.92 [0.66, 1.29]	*0.65 [0.49, 0.86]	0.80 [0.61, 1.05]
**Income**	0.98 [0.90, 1.07]	*0.70 [0.57, 0.86]	0.99 [0.93, 1.06]	0.97 [0.92, 1.02]	0.97 [0.92, 1.03]
**Field center**					
Bronx, NY[b]	1.00	1.00	1.00	1.00	1.00
Chicago, IL	1.19 [0.68, 2.08]	*2.82 [1.19, 6.69]	1.48 [0.93, 2.35]	*0.61 [0.40, 0.94]	*1.88 [1.17, 3.03]
Miami, FL	1.60 [0.73, 3.50]	1.50 [0.55, 4.13]	1.07 [0.59, 1.93]	0.94 [0.51, 1.73]	0.99 [0.59, 1.68]
San Diego, CA	*0.42 [0.19, 0.94]	1.85 [0.66, 5.20]	1.77 [0.94, 3.34]	0.69 [0.41, 1.14]	1.90 [0.96, 3.77]
**Health insurance status**[c]	*2.24 [1.40, 3.58]	1.42 [0.74, 2.74]	1.39 [1.00, 1.94]	1.21 [0.93, 1.59]	*1.46 [1.12, 1.90]
**SASH Social Subscale**	1.27 [0.85, 1.90]	0.95 [0.52, 1.76]	0.85 [0.65, 1.10]	1.18 [0.92, 1.51]	1.06 [0.86, 1.32]
**Alcohol**[d]	0.92 [0.55, 1.54]	0.79 [0.43, 1.45]	0.81 [0.56, 1.17]	0.92 [0.67, 1.27]	0.84 [0.62, 1.15]
**Smoking**[d]	*1.57 [1.06, 2.32]	0.77 [0.40, 1.47]	*1.58 [1.14, 2.19]	1.09 [0.84, 1.40]	0.99 [0.78, 1.26]
**BMI**	*1.04 [1.01, 1.07]	1.01 [0.97, 1.06]	*1.03 [1.01, 1.05]	*1.10 [1.08, 1.12]	*1.10 [1.08, 1.12]

[a]Female as reference group; odds ratios represent effect of being male. [b]Categories with odds ratios of 1.00 reflect the reference group for each variable. [c]No health insurance as reference group; odd ratios represent effect of having health insurance. [d]Dichotomized as current or former smoking/drinking vs never smoking/drinking.
*p<.05
